# Applying Circuit Theory for Corridor Expansion and Management at Regional Scales: Tiling, Pinch Points, and Omnidirectional Connectivity

**DOI:** 10.1371/journal.pone.0084135

**Published:** 2014-01-30

**Authors:** David Pelletier, Melissa Clark, Mark G. Anderson, Bronwyn Rayfield, Michael A. Wulder, Jeffrey A. Cardille

**Affiliations:** 1 Department of Natural Resource Sciences and McGill School of Environment, McGill University, Ste. Anne de Bellevue, Québec, Canada; 2 The Nature Conservancy, Eastern Resource Office, Boston, Massachusetts, United States of America; 3 Department of Biology, McGill University, Montréal, Québec, Canada; 4 Canadian Forest Service (Pacific Forestry Centre), Natural Resources Canada, Victoria, British Columbia, Canada; Cirad, France

## Abstract

Connectivity models are useful tools that improve the ability of researchers and managers to plan land use for conservation and preservation. Most connectivity models function in a point-to-point or patch-to-patch fashion, limiting their use for assessing connectivity over very large areas. In large or highly fragmented systems, there may be so many habitat patches of interest that assessing connectivity among all possible combinations is prohibitive. To overcome these conceptual and practical limitations, we hypothesized that minor adaptation of the Circuitscape model can allow the creation of omnidirectional connectivity maps illustrating flow paths and variations in the ease of travel across a large study area. We tested this hypothesis in a 24,300 km^2^ study area centered on the Montérégie region near Montréal, Québec. We executed the circuit model in overlapping tiles covering the study region. Current was passed across the surface of each tile in orthogonal directions, and then the tiles were reassembled to create directional and omnidirectional maps of connectivity. The resulting mosaics provide a continuous view of connectivity in the entire study area at the full original resolution. We quantified differences between mosaics created using different tile and buffer sizes and developed a measure of the prominence of seams in mosaics formed with this approach. The mosaics clearly show variations in current flow driven by subtle aspects of landscape composition and configuration. Shown prominently in mosaics are pinch points, narrow corridors where organisms appear to be required to traverse when moving through the landscape. Using modest computational resources, these continuous, fine-scale maps of nearly unlimited size allow the identification of movement paths and barriers that affect connectivity. This effort develops a powerful new application of circuit models by pinpointing areas of importance for conservation, broadening the potential for addressing intriguing questions about resource use, animal distribution, and movement.

## Introduction

Forest ecosystems are a complex mosaic of components such as trees, lakes, and wetlands, as well as anthropogenic features such as roads, agriculture, and urban areas. As forest conversion for agriculture and human habitation has continued, protecting the ability of organisms to reproduce in and move among forest fragments has become a major priority in forest conservation [1]–[4]. Connectivity among forest patches can facilitate or constrain movements of abiotic and biotic components of forest ecosystems [5], [6]. Biotic movements of genes, individuals, or populations through a network of forest patches may be critical for the maintenance of ecological and evolutionary processes across multiple spatial and temporal scales: these include, for example, resource acquisition [7]; metapopulation colonization rates [8]; metapopulation extinction rates [9]; seasonal migration [10]; and range shifts in response to climate change [11].

A large and growing number of studies employ network-based connectivity models, in which a landscape is represented as a set of high-quality habitat nodes, with links connecting pairs of nodes if movement is possible between them [12]–[17]. This concept has been used widely to test hypotheses about animal movements and genetic exchange among habitat patches [17]–[21]. Network-based representations can be analyzed to derive meaningful connectivity statistics [22]–[25], though these are often dependent on an assumed graph-theoretic model (e.g., complete graph, minimum planar graph) [19] and are often computer resource-limited as larger areas are considered [26].

In recent years, a new use of graph models to understand habitat connectivity has emerged that conceptualizes a landscape akin to an electrical circuit, with each cell in a raster grid presenting a given “resistance” to movement of modeled organisms [27]–[30]. Foremost among these is the framework implemented in the Circuitscape program [28], [31], which has reshaped the science and capacity for estimating and understanding landscape connectivity. Following its introduction for explaining genetic differences among geographically separate populations of threatened plants and animals [29], Circuitscape has proven useful in a variety of genetic studies, for example for wood frogs [32], boreal toads [33], African elephants [34], golden-headed lion tamarins [35], mountain goats [36], pumas in Brazil [37], lynxes in Canada [38], American martens [39], and humans in ancient societies [40].

Circuitscape’s success in modeling the effects of animal movement at very long time scales has provoked curiosity about its application at finer time scales for management, where movement and distribution are of primary concern [41], [42]. In light of recent successes in connecting resistance-based approaches with movement patterns [38], [43], [44], at least two challenges remain that limit their routine use in landscapes at movement-relevant scales of time and space over large areas. First, because its technical demands increase with the number of land-surface pixels being considered, circuit modeling runs are constrained to limited raster sizes: relatively small areas or large areas at coarsened spatial resolutions [31], [33], [36], [45], [46]. Second, the principal application of landscape circuit analysis has been for point-to-point calculations– for instance, to estimate the landscape contribution to the genetic distance between two populations. While this approach has been proven useful for estimating the resistance distance between particular areas or populations, it remains difficult to generalize from the point-to-point mode into maps showing landscape connectivity across large areas. These limitations inhibit circuit theory’s potential use to identify potential movement corridors, at a fine level of detail spanning a very large area.

To broaden circuit theory’s applicability for corridor expansion and management at regional scales, what is needed to address these limitations is a circuit-based connectivity analysis that is scalable to arbitrarily large areas while presenting a resistance-based assessment at a high pixel resolution. An earlier report by Anderson et al. [47] outlined a tiling approach to produce a continuous map, based on circuit theory, of potential movement paths in multiple directions. The method is intended to allow users to identify hypothesized movement paths, especially to view areas where movement options are constricted. Key features of such “omnidirectional connectivity” maps would be the preservation of a land-cover map’s original spatial resolution while revealing both local movement paths between neighboring habitat patches and regional movement paths that may span the study area.

This study has the following objectives: (1) formalize and refine the tiling of Circuitscape output to form an estimate of omnidirectional connectivity; (2) develop standard assessments to quantify the prominence of seams at the junction of tiles in the current density mosaic; (3) systematically explore the effect of tile size and buffer size on the consistency of model results; and (4) guide the interpretation of these connectivity mosaics to identify areas of high interest for corridor expansion and management.

## Methods

### Study Area: Land Cover and Resistance to Movement

**Figure 1:**
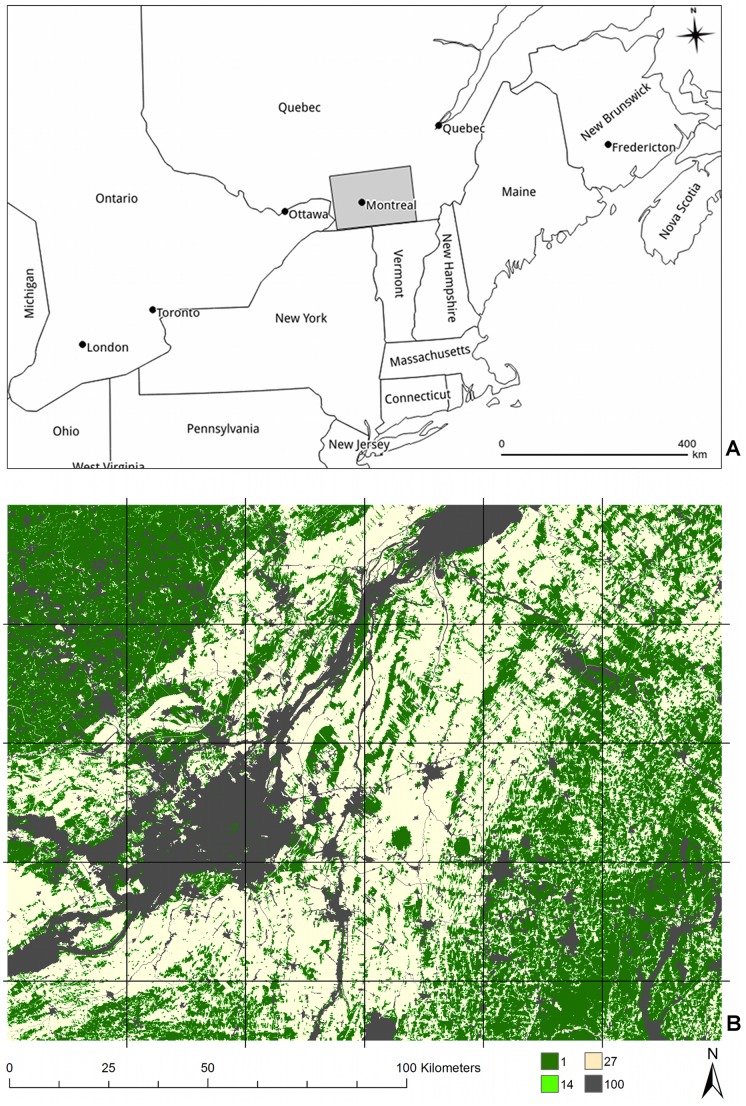
Location and land cover of the Montérégie. (a) Location of the study area centered on the Montérégie region, Québec, Canada. (b) Land-cover map and estimated per-pixel resistance: forest (1) is dark green, vegetated forest buffers (14) are light green, vegetated open areas (27) are beige, non-vegetated areas (100) are dark gray. The grid shows the location of all 1000×1000 pixel tiles.

The Montérégie area is located in Québec, Canada south of the Saint-Lawrence River, and the city of Montréal (Figure 1). Similar to many developed regions with a history of urbanization and agriculture, the region includes a mix of farms, fragmented forests, and intact or re-grown forest surrounding an increasingly urban, populated core. In this area, there is a gradient of forest conversion and perforation from the center of the district toward the east and southwest parts of the region (Figure 1). Land-use data for a 24,300-km^2^ region around the Montérégie was obtained from the Système d'information écoforestière (SIEF; [48]) and the Base de données topographique du Québec [49]. The original SIEF data has a spatial resolution of 30 meters per pixel and is composed of 23 different classes, which we reclassified into four categories: forest; vegetated forest edge; vegetated open areas (e.g., agriculture); and non-vegetated open areas (e.g., urban areas and water).

For Circuitscape to model a landscape as analogous to an electrical circuit, each pixel is assigned a resistance value based upon land cover type. The resistance value represents the relative effort required for a given organism to traverse a pixel on the map, and the map of resistance values is used to derive all the possible pathways for modeled electrical current to traverse the landscape from one point or region to another [25]. For this illustration, we parameterized Circuitscape using resistance values developed by Desrochers et al. [50], who estimated land-cover-specific resistance values for ovenbirds (*Seiurus aurocapilla*) from translocation data: 1 for forest, 14 for vegetated forest edge, 27 for vegetated open areas and 100 for non-vegetated areas.

### Directional and Omnidirectional Connectivity with Circuitscape

**Figure 2:**
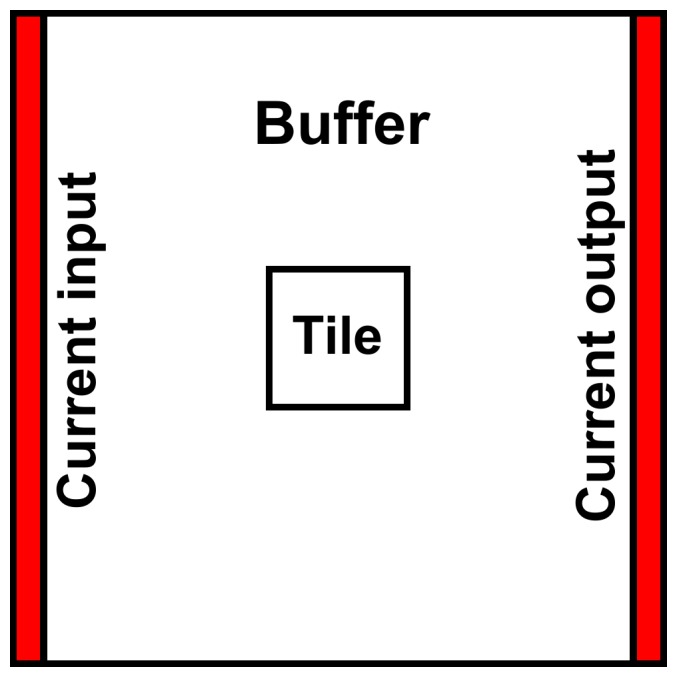
Anatomy of a tile. Creating a tile for a directional run. Circuitscape calculates current density by conducting current through a Circuitscape calculation area formed from a buffer surrounding a tile. Straight, parallel regions allow current to flow through the best paths in the tile; the buffer area is then cropped before assembling the mosaic. Shown is the orientation referred to in the text as an “east-west” run.

To build a connectivity mosaic for the entire study area we partitioned it into a series of overlapping smaller square tiles (Figure 1b). We adapted existing functionality of Circuitscape to create mosaics of “directional” and “omnidirectional” connectivity. To minimize border effects that could be formed by the tiling process [51], we created a buffer around each tile with the surrounding land cover data to form larger and overlapping calculation areas for processing in Circuitscape (Figure 2). For each calculation area, we created regions made of thin, parallel strips one pixel thick along opposing sides, and used Circuitscape to simulate the discharge of current from one side of the calculation area to the other (Figure 2).

**Figure 3:**
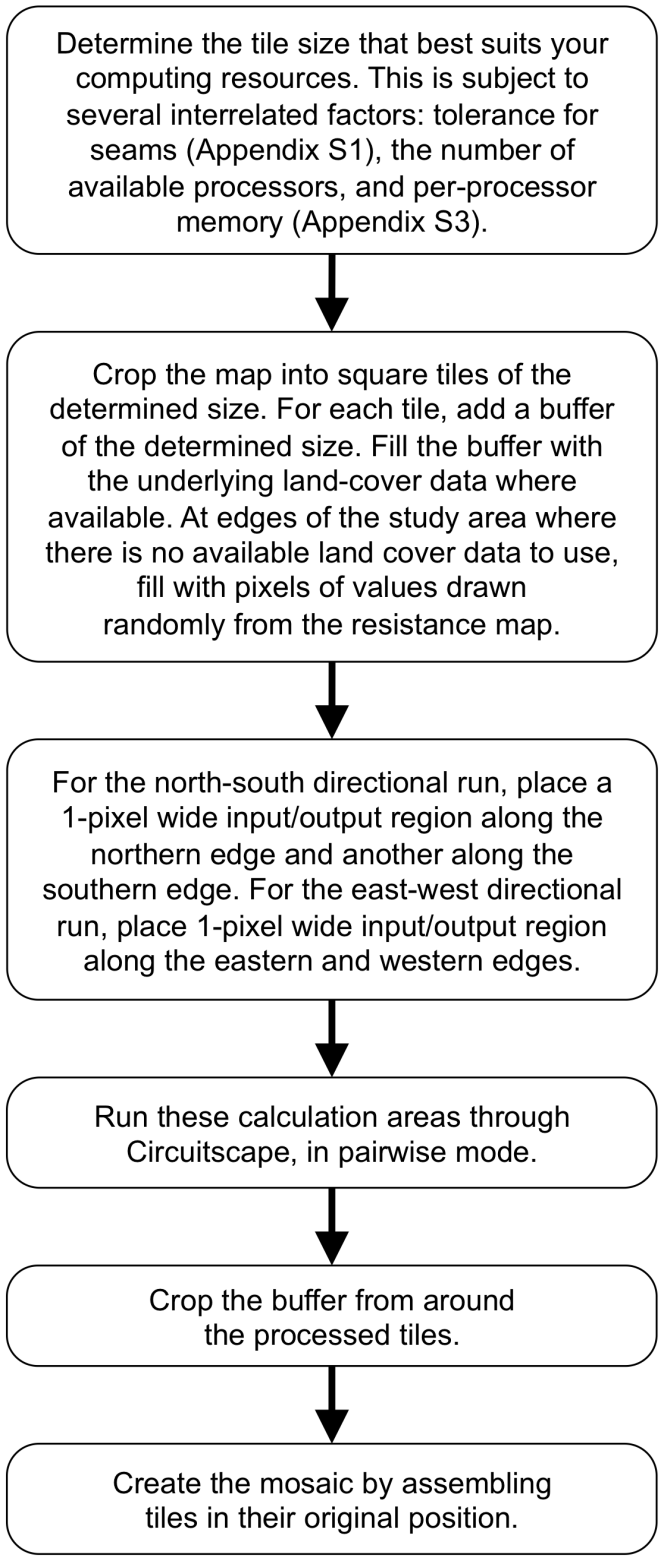
Steps to creating an omnidirectional connectivity mosaic. Summary of the steps required to create an omnidirectional connectivity mosaic.

To calculate the omnidirectional connectivity map for a given tile (Figure 3), we implemented two runs using the pairwise option in Circuitscape [31]: one with parallel vertical input and output regions that forced current horizontally, in the east-west and west-east directions (as in Figure 2), and one with parallel horizontal input and output regions forcing current vertically, in the north-south and south-north directions. After generating the current density maps for the two directional tiles, the buffer area was cropped to retain the original tile’s area. The tiles were reassembled into directional mosaics spanning the entire extent of the original study area. The two directional current density mosaics were combined by multiplication into a single omnidirectional current density mosaic. To test the robustness of our omnidirectional connectivity mosaics, we produced current density mosaics using two other arbitrary oblique directions and explored similarity among the resulting mosaics.

### Mosaic Creation and Analysis

After the runs of individual tiles were completed, they were assembled into a mosaic to form an omnidirectional connectivity map covering the study area. Mosaics were created without any smoothing or manipulations of current density values between the tiles. To understand potential variation in the connectivity mosaic resulting from the choice of tile size and Circuitscape calculation area, we systematically varied these choices with a full 3 by 4 factorial design (Appendix S1). We then compared the 12 resulting mosaics using two measures as described below.

First, we compared mosaics produced with this method by inspecting “seams” at tile edges. As seen in the report of Anderson et al. [47], seams are rows or columns in a given mosaic where values originating in one tile appear next to values taken from an adjacent tile. As such, seams with substantially different values of current density in adjacent rows or columns can reveal the tiling framework of a given mosaic. We defined “seam prominence” as the difference between the current density values for all points along a seam, calculated using Euclidean distance. Given this definition, low values of seam prominence were, in general, a desirable trait of a current density mosaic. Especially prominent seams may not be rooted in real patterns of land-cover composition or configuration. Because current density varies across a landscape according to the underlying land use/land cover patterns, no two adjacent rows or columns have exactly identical current density values. For this reason, seam values in a given mosaic may approach zero, but are not expected to be zero. We performed a two-way repeated-measures ANOVA to explore the relative power of tile size and of Circuitscape calculation area to minimize the seam prominence of a mosaic.

Second, we compared each of the 12 mosaics on a pixel-by-pixel basis to ask whether the choices of tile size and Circuitscape calculation area produced substantially different mosaics representing connectivity in the study area. Using the mosaic with the smallest value of seam prominence (what would informally be called the best mosaic) as the standard, we quantified the difference between it and the other mosaics. To compare two mosaics on a per-pixel basis, values needed to be standardized between them. Our Circuitscape runs used a standard value of 1 volt regardless of the landscape area, so the choice of tile size and Circuitscape calculation area meant that mosaics with identical patterns were formed from different absolute current density values. To compare two mosaics, then, we first transformed the current density values with log10, as recommended for easier visual inspection by McRae et al. [27]–[29]. We then stretched the logged values between 0 and 255, and calculated the per-pixel Euclidean difference between the two mosaics of stretched values. Differences between the same pixel in two different mosaics were expressed as a percentage of the maximum possible (255).

## Results

### An Omnidirectional Estimate of Connectivity

**Figure 4:**
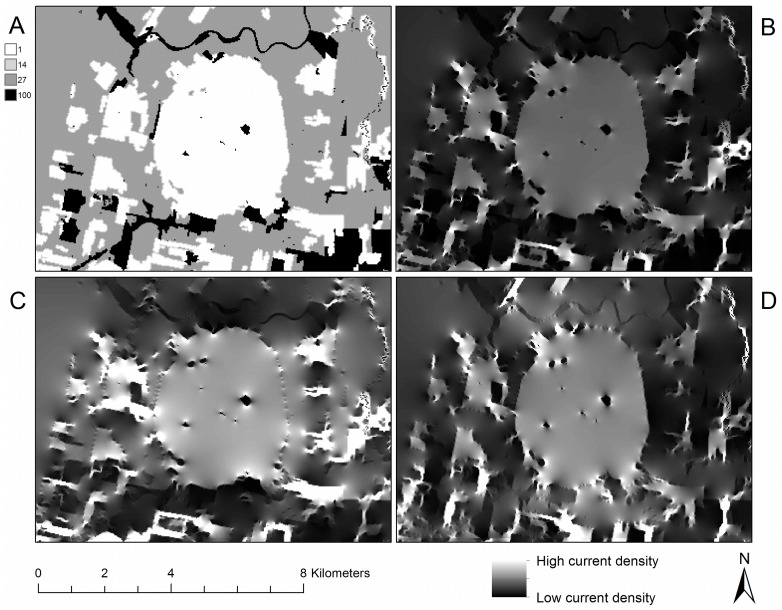
Comparison of directional and omnidirectional mosaics. An omnidirectional connectivity mosaic created using the tiling method, excerpted for a small focal region surrounding Mount Yamaska in the Montérégie. Panel (a): Resistance map. Panel (b): Omnidirectional current density mosaic of the north-south and east-west Circuitscape runs. This was formed by multiplication of the two mosaics in panels (c) and (d), and emphasizes areas where current is high in both orientations. Panel (c): Current density mosaic calculated in the east-west and west-east directions. Panel (d): Current density mosaic calculated in the north-south and south-north directions.

The omnidirectional connectivity mosaic (Figure 4) illustrates the current flow, given the arrangement of land-cover resistance values, through each pixel of the map in multiple directions. Current flows around obstacles of high resistance in favor of flow through low- and medium-resistance land cover types (Figure 4a), responding to the composition and configuration of the landscape. In the east-west directional mosaic (Figure 4c), the landscape is configured in such a way that travel through the mountain’s forests is heavily favored. The east-west directional mosaic illustrates several areas in the east-west direction where a substantial amount of current is forced to travel across non-forested areas (compare open areas west of the forested circular feature of Mount Yamaska in Figure 4a with Figure 4c). This reveals clear variation in the expected use of the landscape, given the resistance parameters modeled here, by animals travelling in this direction. In the north-south direction (Figure 4d), the current appears in paths that tend north and south. The choice of which orientation angle to use when creating tiles for the study area produced no discernable difference in the resulting current density mosaics (Appendix S2). Using either of two other orientations, as well as incorporating the other orientations into a mosaic by blending it with tiling in the cardinal directions, did not discernibly affect the spatial patterns seen on the current density mosaic. This confirmed our experience that north-south and east-west runs were sufficient to capture the spatial variation in current density without the need to incorporate a suite of other tiling angles.

### Shadow Effects in High-resistance Areas

**Figure 5:**
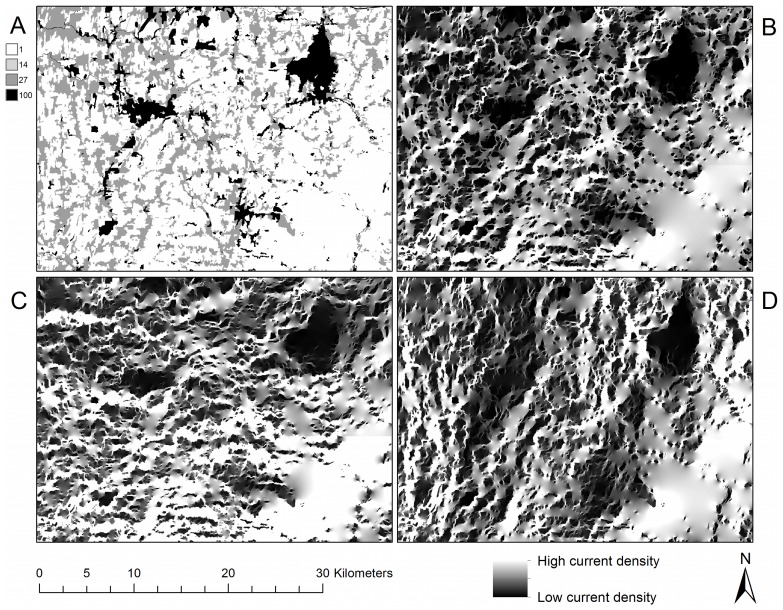
The effect of the configuration and composition of the landscape on current flow. Shadow effects of high-resistance areas in directional and omnidirectional connectivity mosaics for a region in the southeastern portion of the study area. Panel (a): Resistance map. Panel (b): Omnidirectional current density mosaic. Panel (c): Current flow in the east-west and west-east directions. Panel (d): Current flow in the north-south and south-north directions.

High-resistance areas produce important secondary effects in omnidirectional connectivity mosaics (Figure 5). Contiguous regions of high-resistance land cover types–here cities had the highest per-pixel resistance–produced a “current shadow” in complex shapes around the high-resistance pixels. For example, in the city located in the northeast part of the image in the east-west directional flow (Figure 5c), the shadow is larger than the city itself and visible well beyond the city’s pixel limits. This appears to be driven by the city’s configuration: the wider (eastern) part of the city casts a bigger shadow. Similarly, the north-south directional flow (Figure 5d) revealed a more substantial shadowing effect, for those obstacles (cities, in this case) that were wider in the dimension perpendicular to that of the modeled flow. The omnidirectional connectivity mosaic (Figure 5b) both blends and enhances these shadowing effects in its representation of connectivity in all directions. The north-south shadows of Figure 5d were still present in the omnidirectional connectivity mosaic (4b), though at a lesser intensity and different spatial structure. Overall, the shadows of current flow cast by cities were linked not only to the size of the high-resistance areas, but also to their configuration and placement in the landscape.

### Uneven Flow of Current in the Landscape

**Figure 6:**
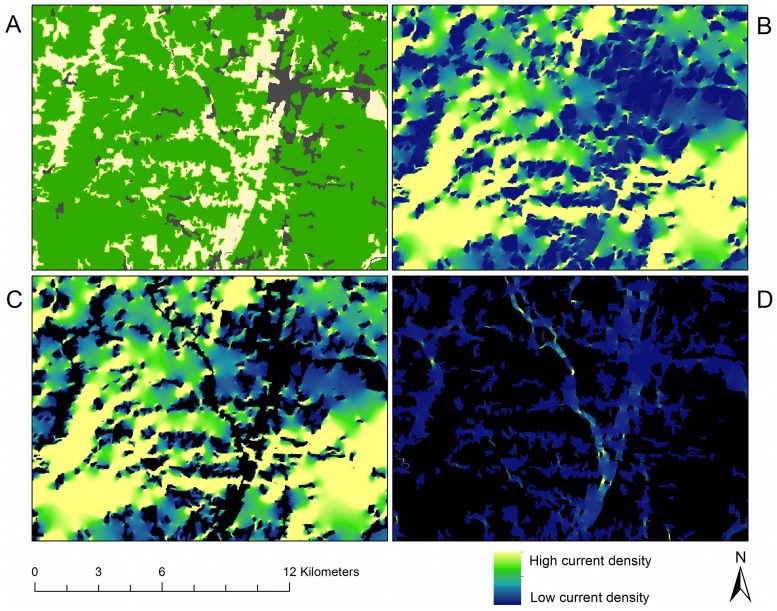
Uneven use of the landscape. Omnidirectional connectivity for a small focal region seen in the lower middle part of Figure 5. Panel (a): Resistance map: forest is green, open vegetated areas are beige and non-vegetated open areas are dark gray. Panel (b): Current density mosaic formed from north-south and east-west Circuitscape runs. Panel (c): Omnidirectional current density mosaic shown only in forest areas, other areas have been masked. Not all forest, despite its low resistance, is equally important to omnidirectional current flow. Panel (d): Omnidirectional current density mosaic with forested areas masked, revealing high-current areas where creation of corridors might be prioritized.

Given the resistance settings of this illustration, forest pixels have, in general, high current density in the omnidirectional mosaic of the Montérégie (Figure 6). Yet it is clear that current density also differs substantially within those forests. Tracing along the eastern edge of the current density mosaic (Figure 6b) shows the uneven distribution of current passing through forests, which is particularly low in and around the area delineated by the outer edges of the high-resistance city. The substantial variation in current density suggests that not all forest in this landscape is equally important for providing connectivity; rather, the Circuitscape algorithm indicates that a complex interplay of land-cover composition and configuration influences the contribution to landscape connectivity provided by a given area.

Outside forests (Figure 6d), areas of high current density indicate where the demand for access from one forest patch to another is so great that current jumps across open areas despite their high resistance. Like uneven flow in forests, non-forest current flow is not uniform, but rather favors those areas where land-cover composition and configuration force flow away from low-resistance forest pixels. High current flow in non-forest areas corresponds to open areas that are not currently part of an entirely forested corridor, but which, according to the omnidirectional model, could form part of heavily traveled routes (Figure 6d).

### Mosaics of Current Density in the Montérégie

**Figure 7:**
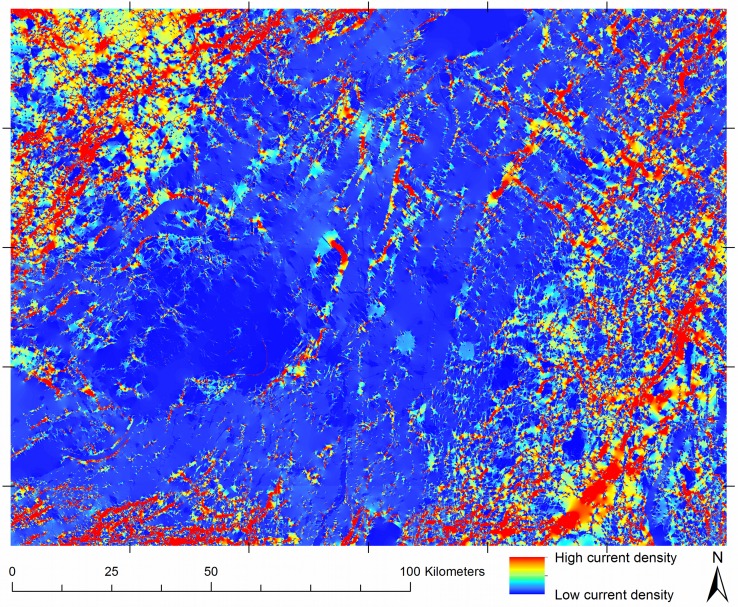
Complete omnidirectional connectivity mosaic of the Montérégie. Omnidirectional mosaic built from 30 tiles of 1000×1000 pixels with 200% buffers. The image represents the multiplication of the two directional mosaics as described in the text. The location of the tiles, shown with exterior grid marks, indicates where tile seams are located.

Figure 7 shows a mosaic representing omnidirectional forest connectivity in the Montérégie region. Produced from 30 tiles, the mosaic is clearly dominated by the characteristics of the amount and location of forest, as would be expected from the resistance values used. In general, current density is highest in forest pixels, indicating that given the resistance values and the spatial configuration of land covers in the study area, current flows through forest whenever possible. The connectivity mosaic estimates that in general, forests located near urbanized areas are likely to be less frequently used for movement.

The choices of tile size and buffer size used to create the calculation area affected the prominence of seams in strongly predictable ways (Table 1). There was a significant effect across three tile sizes [F = 62.77, DFn = 2, DFd = 78, p = 5.67E-17*] and four buffer sizes [F = 122.06, DFn = 3, DFd = 117, p = 7.06E-36*]. There was no significant interaction between the factors (Figure S1 in Appendix S1). Using either a larger tile size or larger buffer to create the Circuitscape calculation area served to produce mosaics with less prominent seams. The strength of each factor is of the same order of magnitude (Table 1), meaning that both factors can contribute substantially to reducing seam prominence.

**Table 1:** Effect of tile size and buffer size.

Effect	DFn	DFd	F	p	p<0.05	ges
**Size**	2	78	62.77	5.67 E-17	*	0.0610
**Buffer**	3	117	122.06	7.06 E-36	*	0.0879
**Size:Buffer**	6	234	2.10	5.41 E-02		0.0020

ANOVA showing effects of tile size (“Size”) and buffer size (“Buffer”) on seam prominence. The Generalized Eta-Squared measure of effect size (“ges”) indicates that the two factors have similar leverage on decreasing a mosaic’s seam prominence. The p score also indicates that the interaction of tile size and buffer size is not significant at the 0.05 level, and that its leverage on seam prominence is minimal in comparison to the factors.

**Figure 8:**
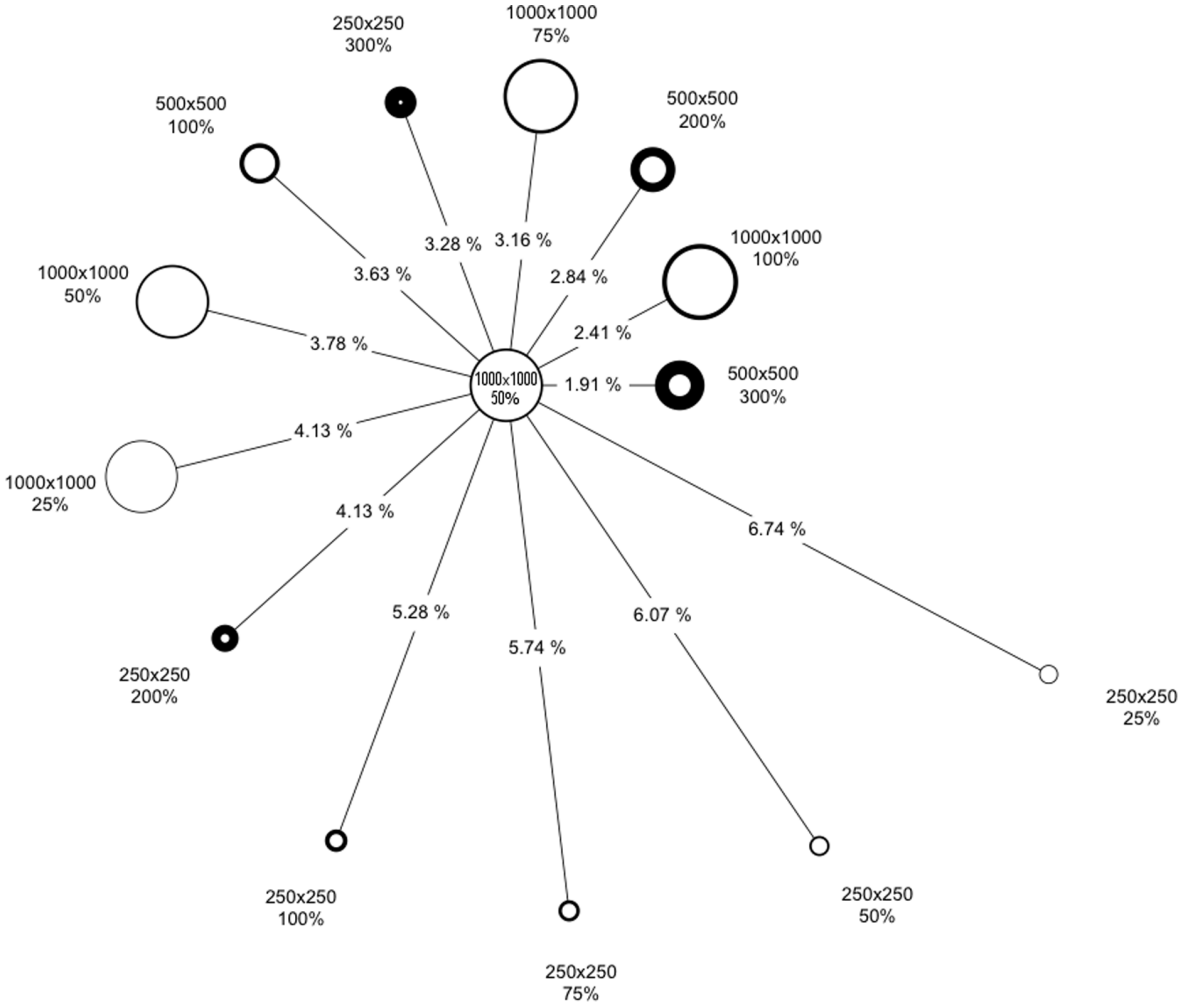
Per-pixel comparison among connectivity mosaics. Relative difference between the results of a suite of mosaics. The length of each line represents the difference between each mosaic and the mosaic shown in Figure 7; the more different two mosaics are, the farther apart they are placed. The size of a circle denotes the tile size in each mosaic (bigger circles means bigger tiles). The thickness of a circle’s border denotes the size of the buffer used to create the calculation area (thicker means larger buffers).

Figure 8 shows per-pixel differences between mosaics made using different tile sizes and buffer sizes in the Monteregie region. With respect to the mosaic having the smallest value of seam prominence (what would informally be called the best mosaic), the most similar results had the largest buffer sizes (500×500 tiles with a 200% buffer), the largest tile sizes (1000×1000 tiles with a 75% buffer, 1000×1000 tiles with a 50% buffer), or both (1000×1000 tiles with a 100% buffer). Despite the substantial differences among tiling parameters, current density mosaics were quite similar, with the major differences among mosaics being the difference in seam prominence. Given that the seam prominence could be precisely tuned with a combination of buffer size and tile size, different demands of computer memory and processor speed (Table S1 in Appendix S3) can guide parameter choices for the construction of a current density mosaic for a study area.

## Discussion

The ability to create an omnidirectional connectivity mosaic across a large area addresses the pressing challenge of producing “maps of areas important for connectivity” [52]. It substantially extends the reach of circuit theory’s application to landscapes by enabling a seamless, arbitrarily large omnidirectional connectivity map at the full resolution of the input land cover information. Our prototype analysis produces a quantitative mapping of core habitat areas and possible movement pathways through the landscape. Somewhat surprisingly, preliminary testing for this study indicated that two directional runs appeared to capture the meaningful spatial variability in current density; incorporating additional directions did not result in discernibly different mosaics. The mosaics indicate high-flow regions that can be integral to landscape connectivity, and can be difficult to identify in non-circuit software or through point-to-point calculations of resistance distance.

The development of this omnidirectional connectivity mosaic suggests many hypotheses that can now be quantitatively tested, potentially for application at fine scales of time and space. For example, pinch points [28] in these connectivity mosaics could be used to target monitoring efforts, for example to prevent unauthorized human activities such as illegal logging. We note that the digital designation of a given connectivity value does not imply certainty of where a given organism will travel. Nevertheless, we can hypothesize that a well-parameterized connectivity mosaic provides information for ranking possible movement paths or working towards the producing of statistical likelihoods of animal movement through these paths, as in Walpole et al. [38], [53]. Testing of connectivity mosaics against pertinent observation data sets could also help refine the resistance values chosen for a given organism and application. It has already been demonstrated that across long time scales at which genetic mutations can be measured, resistance distance is strongly correlated to the differences between populations [28], [29], [42]. It remains to be seen if the omnidirectional current density mosaics developed here will show a similar strength in fitting animal movement data on much shorter time scales.

Using the connectivity mosaic (Figure 6, Figure 7), potential locations for corridor expansion and priority management can be identified. Avenues of high current density suggest that the organisms under study (given that the chosen resistance estimates adequately represent true movement characteristics) are likely to pass through them as they move through the landscape. These avenues may include a combination of habitat coded as optimal and as sub-optimal. Given the results obtained using this method, the forested portions of high-current pathways could be considered high-priority areas for corridor management and conservation. Likewise, for the parts of an identified high-current pathway that are not forested, these tools indicate potential high-priority locations for forest corridor expansion for conservation planning (Figure 6). To us, the identification of locations for habitat expansion seems an especially powerful aspect of this work: animals are well known to dwell in or pass through non-optimal habitat [54], [55], and this method might help reveal where that is most likely in a given landscape. It is hoped that the approach outlined here can contribute to ongoing work in detecting barriers [56], the evaluation of connectivity in protected lands [57], and efforts to increase connectivity in adaptation to climate change [58].

The omnidirectional connectivity mosaic indicates that although per-pixel resistance values may be uniform for a given land cover type when stored in a GIS, differing configurations of land cover can strongly affect the estimated movement paths of organisms through these environments. Given a landscape’s patterns of land cover composition and configuration, not all habitat of a given land cover type is likely to present the same ease of movement for organisms. This can be due to barriers in the immediate area surrounding a given pixel [27] as well as more distant “shadow” effects viewed in this study. Although that conclusion is not surprising, in our experience it is difficult or impossible to identify the specific pathways revealed for an area like the one shown in Figure 6a, except through the careful application of circuit theory as seen here.

More generally, depending on the size of the tiles and buffers used to create the calculation area (Figure 2) for this method, seam lines between tiles may remain visible when a mosaic is assembled for a study area. Prominent seams, which in our opinion are detrimental to the assembly of these tiles into mosaics, can be greatly reduced by increasing the tile size or the buffer size used to create the Circuitscape calculation area. Yet increasing the buffer size, or decreasing the tile size, has the effect of increasing either the processing cost of each tile, the number of tiles needed to cover a large area, or both. Although the processing in Circuitscape of calculation areas of 5000×5000 pixels demanded substantial memory (Appendix S3), there were very few detectable seams in the resulting mosaic.Given the rapid proliferation of studies based on isolation by resistance, it is likely that the development of resistance maps is in its infancy. Notably, the connectivity mosaics produced here are reminiscent of the “resistant kernel” approach developed by Compton et al. [59], which is a hybrid of kernel density and resistance calculation methods. That study produced maps computed to be omnidirectional, showing a continuous model of connectivity amongst selected dispersal source habitats. Their approach creates a dispersal map by summing multiple estimates from the perspective of many discrete habitat pixels, rather than through the directional sweep across a tiled map as presented in our implementation. Circuit theory connectivity models are generally seen to be robust to coarsening of input data [53], which can enable computation across very large areas at coarse pixel resolution [28], [57]. Nevertheless, we see it as noteworthy, for later fine-scale interpretation, that our method creates connectivity mosaics of potentially immense size from tiles that retain their full initial spatial resolution. This feature may increase the applicability of circuit-based connectivity analyses for organisms that interact with the landscape at fine spatial scales due to restricted mobility or habitat requirements.

Compared to the many methods that can be used to quantify the characteristics of a patch-based analysis represented with nodes and links [16] there is a paucity of strategies to analyze the results of continuous connectivity maps [60]. These strategies are in active development [57]–[59], and include the detection of barriers that impede movement [56]. Despite the relative ease with which pinch points can be identified visually on current maps produced by Circuitscape, there are currently no clear guidelines on how one would systematically determine whether an area is a pinch point, or whether a single current value threshold might reliably identify pinch points across a large set of tiles. We propose two avenues to explore for extracting more information from our connectivity mosaic, based on the composition of the current density mosaic and another on its configuration. For broad compositional comparison, one might consider a brightness-based assessment, in which the histograms of current density might be compared across tiles. A second possibility might reinterpret the mosaic as a vector set of nodes and links traced over high current density paths and pinch points, and calculate well-developed network statistics on the resulting graph.

## Conclusion

With increased interest in forest sustainability, it can be expected that there will be increasing focus on measureable, intercomparable characteristics of heterogeneous forest landscapes; these characteristics of interest are certain to include the connectivity within and between forests and other land covers for the movement of animals of different sizes and traits. The continuing improvement and miniaturization of animal monitoring tools suggests that animal movement will be able to be tracked with increasing precision and regularity. From radio-collar animal data, for example, movement trajectories can be computed; using a complementary connectivity map, we will have an improved capacity to hypothesize and analyze the reasons for chosen pathways [38], [53]. These advances will allow a deeper exploration of the movement patterns of an increasing number of animals, and their comparison to assessments of landscape patterns such as those presented here.

Following the approach we developed, it is possible to create omnidirectional connectivity mosaics representing very large areas. Across a wide range of choices of tile sizes and buffer size when creating the Circuitscape computation areas, mosaics were nearly identical, with the identifiable differences among mosaics being the prominence of seams at tile junctures. Seam prominence is highly dependent on choices of tile size and buffer size. Given enough memory and processor power, seam prominence can be decreased in predictable ways until seams are no longer a complicating factor in mosaic creation. The ability to create arbitrarily large mosaics providing a comprehensive view of connectivity should be of considerable use to researchers and practitioners working over broad spatial areas in a variety of different domains.

## Supporting Information

Appendix S1 **Understanding seam prominence.**
(DOCX)

Appendix S2 **Considering non-cardinal directions in mosaic creation.**
(DOCX)

Appendix S3 **Processing time and memory usage.**
(DOCX)
