# Supplementary material for: Applying Circuit Theory for Corridor Expansion and Management at Regional Scales: Tiling, Pinch Points, and Omnidirectional Connectivity
Source: PLoS One. 2014 Jan 30;9(1):e84135. doi: 10.1371/journal.pone.0084135 (PMC3907452; doi:10.1371/journal.pone.0084135)
Supplement: Appendix S1 — Understanding seam prominence. (DOCX) [file pone.0084135.s001.docx]

# Appendix S1: understanding seam prominence

Seam prominence can be used to understand the differences among mosaics created for a study area with different settings of tile size and buffer size. Graphs of current density (Figure S1 in Appendix S1) along an arbitrarily chosen seam from Figure 7 in the manuscript show samples of seam prominence in mosaics for the same area. The panels display values from a line of pixels from the current density mosaic on each side of a tile border, with each side taken from a different tile processed with Circuitscape as described for the tiling process. A high difference in current density along the seam (for example, the run with 250x250-pixel tiles with 50% buffer in Figure S1 in Appendix S1) is revealed in lines spaced far apart, and indicates a prominent seam. Lines that are very close (for example, the 500x500-pixel tiles with 200% buffer in Figure S1 in Appendix S1) denote a smooth seam transition between two tiles in the given mosaic. Because current density varies from row to row and from column to column across a mosaic, lines are not expected to overlap perfectly for any setting of tile size and buffer size.

For a single seam, its “current density difference” can be defined as the square root of the mean squared difference in current density values for every pair of pixels on each side of the seam. In this illustration the mosaic’s “seam prominence” is calculated by averaging the current density difference for 40 seams on a 1000x1000 tile size mosaic. The same seam locations are used for mosaics built using smaller tiles, allowing comparisons amongst mosaics of different tile sizes. A compilation of these values is shown in Figure S2 in Appendix S1, with mosaics built from bigger tile sizes and buffer sizes showing noticeably lower seam prominence scores.

As described in the manuscript, changing the tile size and buffer size used to create the Circuitscape calculation area can control a mosaic’s seam prominence. To create a smooth mosaic with low seam prominence, users of this method should, in general, use large tile and buffer sizes when creating the Circuitscape calculation area of each tile. For larger tile sizes, the total number of tiles required to cover the study area is smaller, which also reduces the total number of seams that form the mosaic. However, larger tiles with large calculation areas require substantially more physical computer memory to process, and the decrease in seam prominence comes at the expense of greatly increased processing time (see Appendix S3).

## Figures

**Current density difference for a seam in a mosaic**


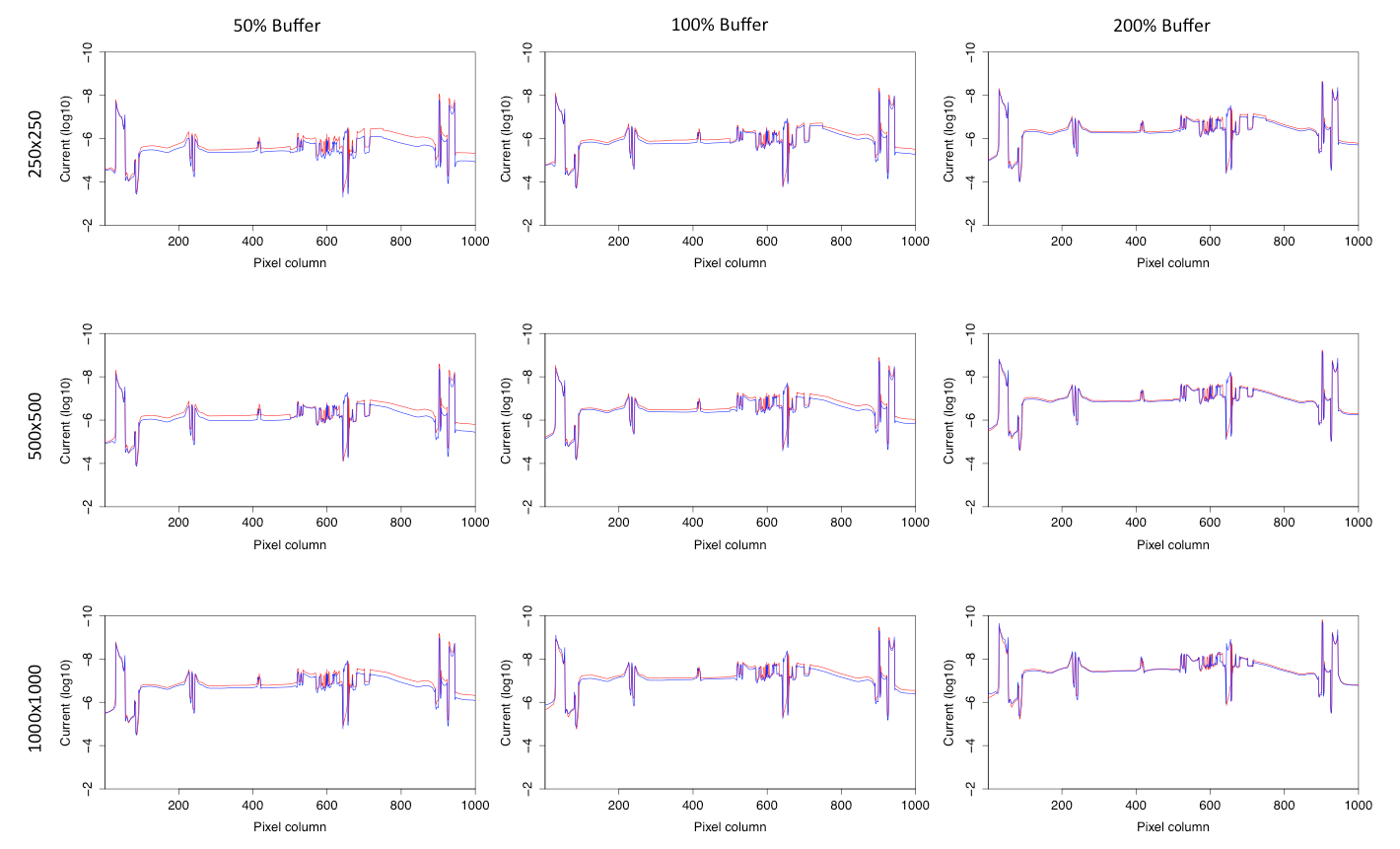


Figure S1: The current density difference of an example seam, as affected by tile size and buffer size. For larger choices of tile size and/or buffer size used to create the Circuitscape calculation area, the difference between each side of a tile seam is smaller. A vertical seam is shown, with the red line representing the west side and the blue line the east side.

**Seam prominence**


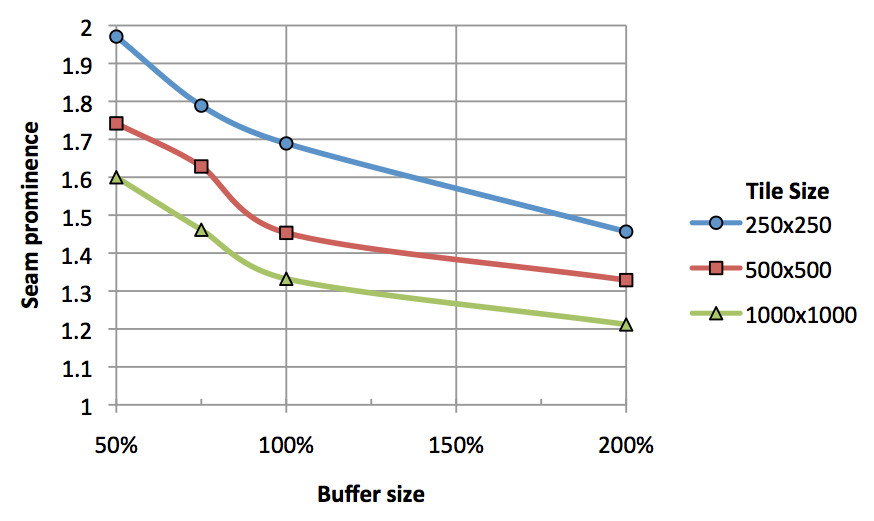


Figure S2: As the buffer size and tile size used to create the mosaic increases, the seam prominence of the mosaic decreases.
