# Supplementary material for: Applying Circuit Theory for Corridor Expansion and Management at Regional Scales: Tiling, Pinch Points, and Omnidirectional Connectivity
Source: PLoS One. 2014 Jan 30;9(1):e84135. doi: 10.1371/journal.pone.0084135 (PMC3907452; doi:10.1371/journal.pone.0084135)
Supplement: Appendix S2 — Considering non-cardinal directions in mosaic creation. (DOCX) [file pone.0084135.s002.docx]

# Appendix S2: considering non-cardinal directions in mosaic creation

### Method to consider more directions

To consider non-cardinal directions when creating a mosaic using the method presented in this manuscript, the grid of input and outputs must be rotated over the resistance map. We used the ArcGIS Fishnet command to create a tiling framework oriented at arbitrary angles (one with tiles rotated -22.37 degrees from horizontal and a second with tiles rotated +16.08 degrees), then created tiles from the resistance map data for the angled study areas. We inserted input and output regions on each sides of the rotated tile as presented in the manuscript (manuscript Figure 2). The rotated tile raster files had a larger spatial extent than the tiles in the manuscript, but to insure that the area of the tiles where current was spread was the same as the tiles used in the manuscript, the four corners were filled with NODATA to keep the same total valid pixel count as the original tiles (Figure S1 in Appendix S2). The combination of multiple directions was done using the mean current value of all directional pairwise runs for each pixel.

### Calculating the differences between different combinations of directions

The difference between all combinations of directions were calculated for a single tile using the same Euclidean difference method we used to calculate the current density differences for seams (Appendix S1) while using the spatial extent of the tile from the manuscript found in the same location (Figure S2 in Appendix S2).

### Results

The differences between all combinations of directions are numerically small (Table S1 in Appendix S2) and visually difficult to distinguish (Figure S3 in Appendix S2). Figure S3a in Appendix S2 shows a tile processed with a pairwise run at -22.37° and a perpendicular run at 67.63°, for a total of two directional runs. These correspond conceptually to the “north-south” and “east-west” runs of the manuscript that were combined to form an omnidirectional connectivity mosaic. Figure S3b in Appendix S2 shows a similar result obtained via pairwise runs perpendicularly at 16.08° and 106.08°. Figure S3d in Appendix S2 shows the combination of all 12 available computed directions: the mosaic presented in the manuscript and the two rotated runs presented in this appendix.

### Difference between the manuscript tiles and the rotated tiles

The omnidirectional tile described in the manuscript and the two rotated tiles are very similar. According to Table S1 in Appendix S2, when compared to the manuscript tile (Figure S3c in Appendix S2), the tile rotated counter-clockwise differs by 1.29% and the tile rotated clockwise differs by 1.06%. Both tiles are visually indistinguishable from the manuscript mosaic.

###

### Effect of incorporating extra directions

The current density values of the tiles do not change substantially when more directions are incorporated. According to Table S1 in Appendix S2, the combination of all 12 computed directions is only 0.67% different from the manuscript tile. These runs are similar enough to conclude that adding more directions does not change the resulting omnidirectional mosaic, and is not worth the considerable increase in processing time required.

### Figures

**A rotated tile computation area**


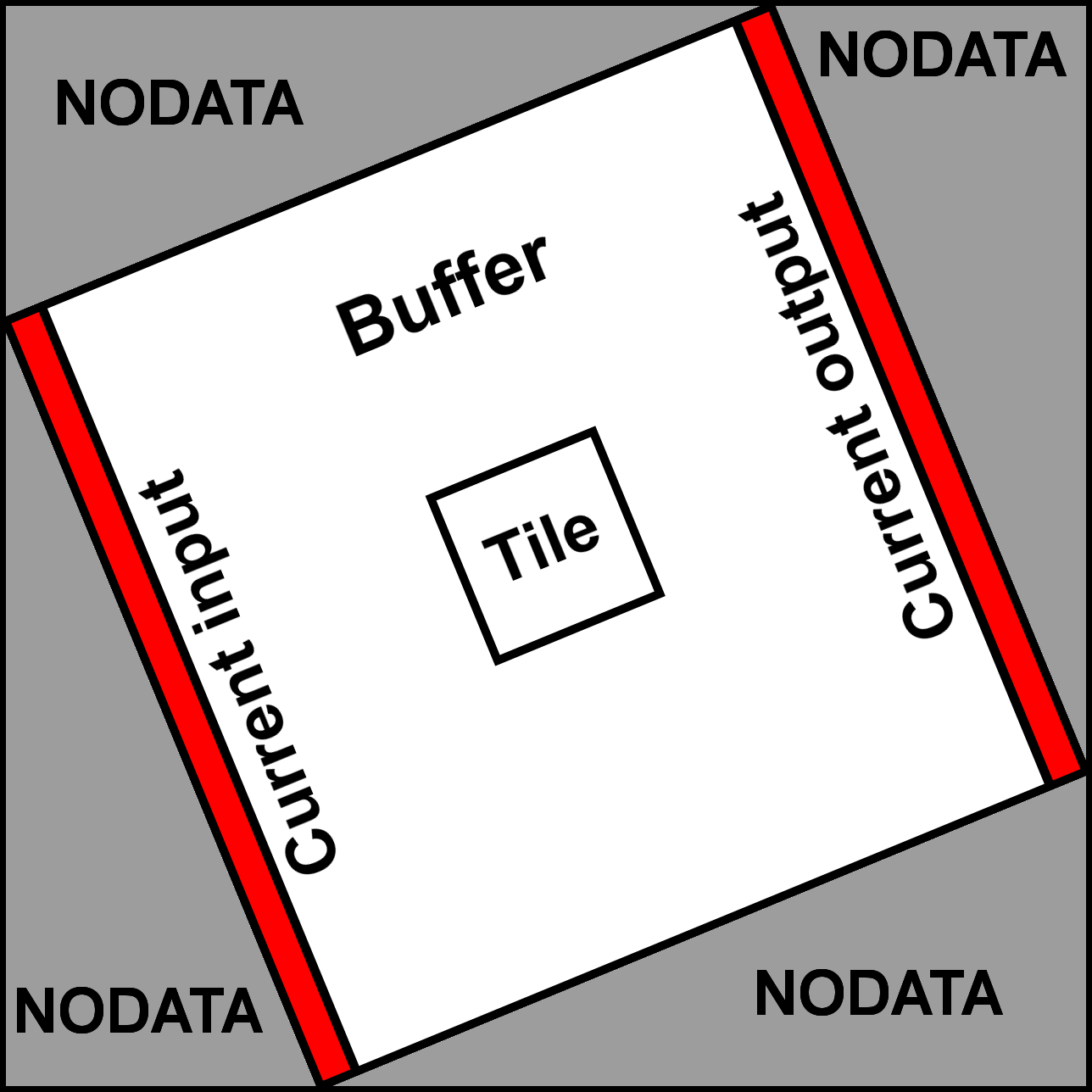


Figure S1: Example of a rotated tile with input and output regions.

**Location of the rotated tile calculation areas**


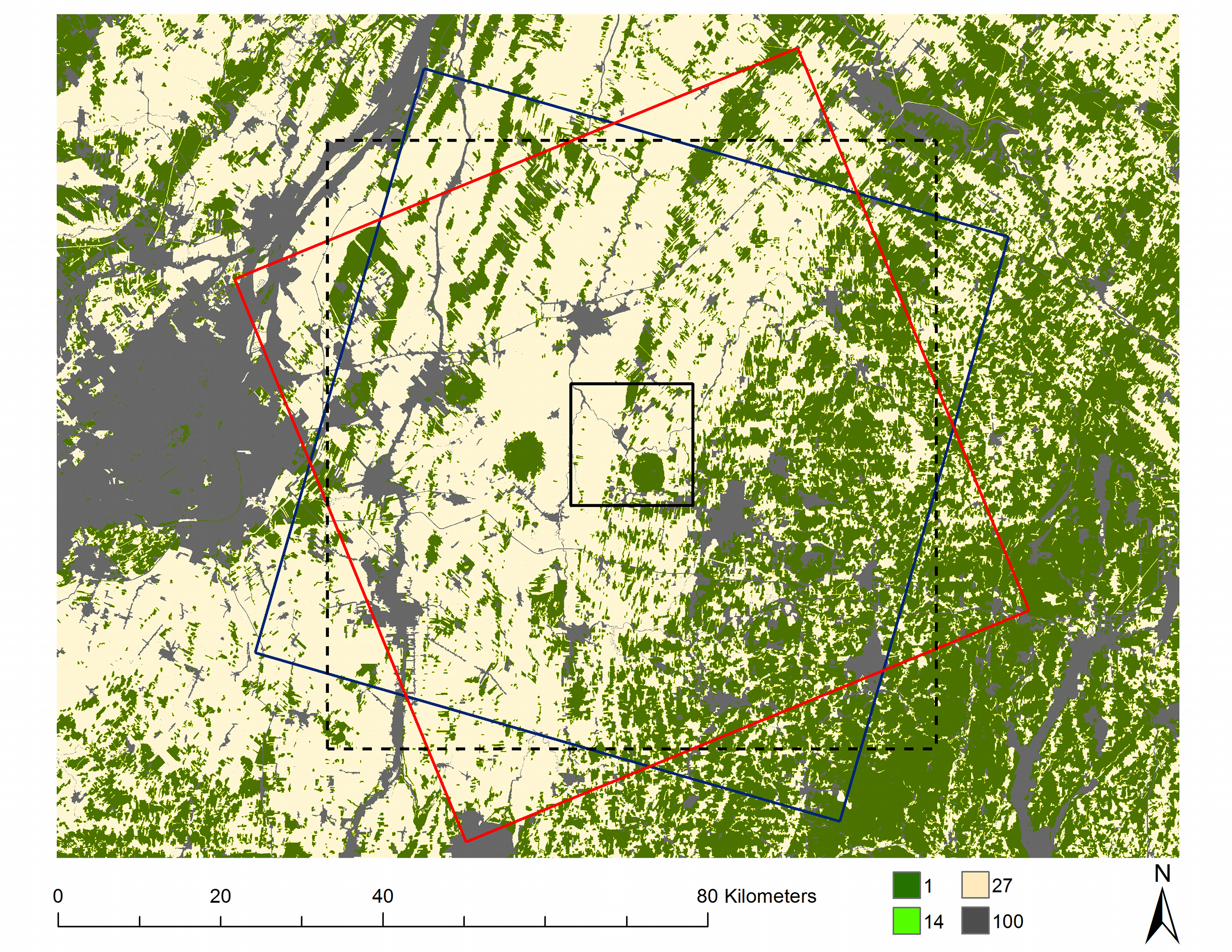


Figure S2: The input and output areas for the rotated tiles. The black outline shows the area of a 500x500 pixel tile. The dashed black outline shows the calculation area of the 500x500 pixel tile with a 200% buffer. The blue outline shows the calculation area of the 16.08° rotated tile and the red outline shows the calculation area of the -22.37° rotated tile.

### Comparison of the rotated tiles


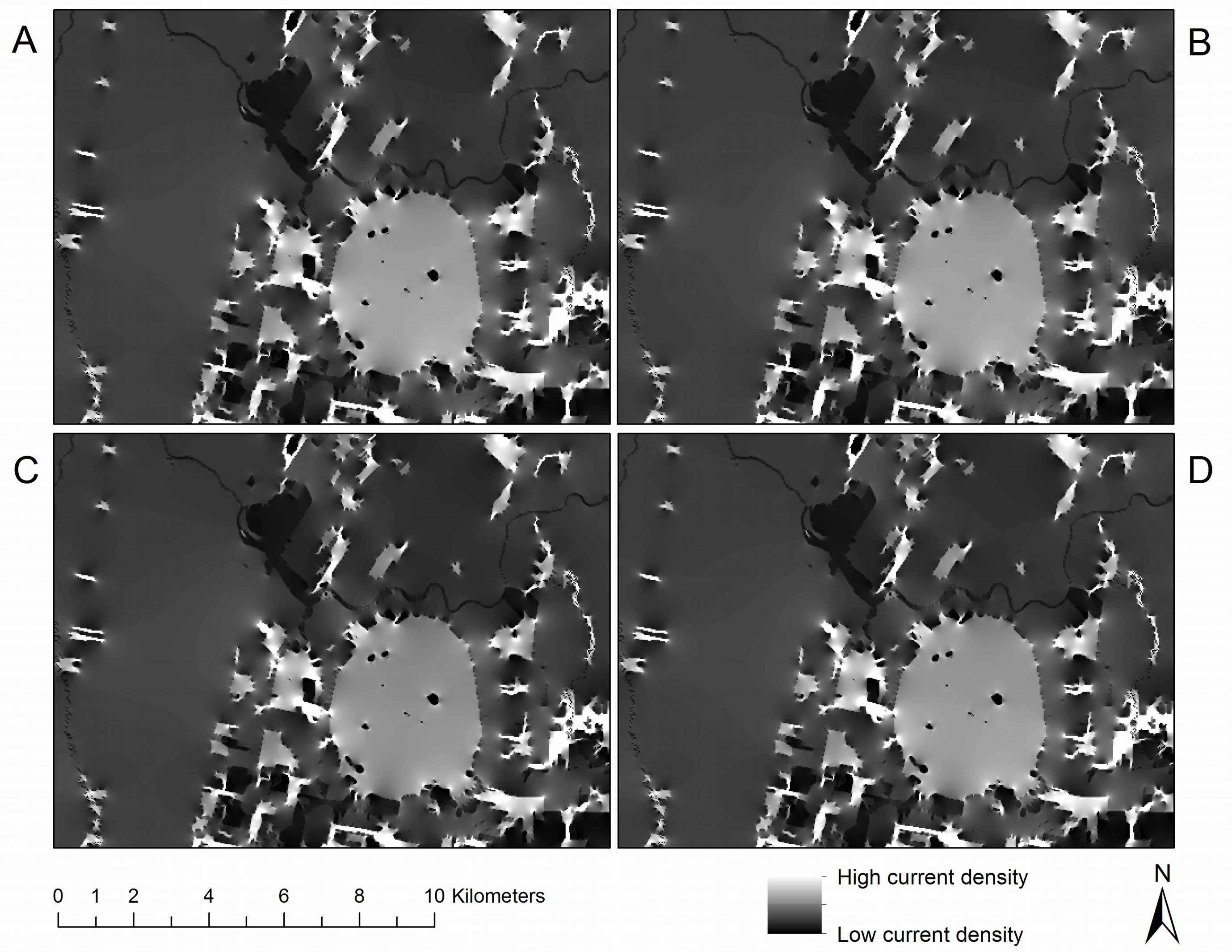


Figure S3: Panel (a): tile rotated -22.37 degrees. Panel (b): tile rotated 16.08 degrees. Panel (c): the non-rotated tile cropped from the manuscript Figure 7 mosaic. Panel (d): all 12 directions combined.

### Tables

**Differences between direction combinations**

| **Comparison pair** | | **Difference (%)** |
| --- | --- | --- |
| ^*^4 directions, 0° | 4 directions, -22.37° | 1.29 |
| ^*^4 directions, 0° | 4 directions, 16.08° | 1.06 |
| ^*^4 directions, 0° | 12 directions | 0.67 |
| 12 directions | 4 directions, -22.37° | 0.77 |
| 12 directions | 4 directions, 16.08° | 0.64 |
| 4 directions, 16.08° | 4 directions, -22.37° | 1.24 |

Table S1: Percentage of difference between several combinations of directions. Tiles marked with a superscript asterisk (*) refer to the mosaic displayed in the manuscript, Figure 7.
