# Supplementary material for: Applying Circuit Theory for Corridor Expansion and Management at Regional Scales: Tiling, Pinch Points, and Omnidirectional Connectivity
Source: PLoS One. 2014 Jan 30;9(1):e84135. doi: 10.1371/journal.pone.0084135 (PMC3907452; doi:10.1371/journal.pone.0084135)
Supplement: Appendix S3 — Processing time and memory usage. (DOCX) [file pone.0084135.s003.docx]

# Appendix S3: processing time and memory usage

### Processing time and memory usage

The ideal combination of tile size and buffer size when creating the Circuitscape calculation area varies depending on the needs of the user and the available computing power. The prominence of seams on the connectivity mosaics made with the tiling method decreases when large tiles and calculation areas are used (Figure S2 in Appendix S1). However, this decrease in seam prominence comes at the cost of increased processing time and memory usage (Figures S1 and S2 in Appendix S3). The processing time of Circuitscape increases linearly with the total pixel count of the resistance map (Figure S3 in Appendix S3). The calculation areas of each tile overlap each other, and as the calculation areas size increases, the amount of overlap keeps increasing. With a buffer size of 100%, each pixel in the resistance map will be present on the calculation area of 9 tiles. If the buffer size is doubled to be 200% of the tile size, each pixel of the resistance map will be present on the calculation area for 25 tiles. This increase in calculation area size for each tile means that a lot more surface has to be processed in Circuitscape when using larger buffers.

For example, a connectivity mosaic made for a study area of exactly 3000x3000 pixels would have 9 tiles of 1000x1000 pixels. With a buffer size of 50%, each tile would be surrounded by a buffer of 500 pixels, for a total calculation area size of 2000x2000 pixels. This results in a total surface of 36 million pixels (9 tiles x 2000 pixels x 2000 pixels) that needs to be processed by Circuitscape. Increasing the buffer size to 100% means that each of the 9 tiles would have a calculation area of 3000x3000 pixels, for a total of 81 million pixels. This results in a total calculation area that has more than twice the surface of the example using a buffer size of 50%, and 9 times the surface of the original resistance map.

Depending on the combinations chosen for tile size and buffer size, mosaics may have near-identical values of seam prominence, but require different amounts and types of computing resources. As shown in Figure S2 in Appendix S1, the seam prominence of the 250x250 tiles with a 200% buffer (henceforth abbreviated as *tile size@buffer size*; 250x250@200%) mosaic and the 1000x1000@75% mosaic is the same at 1.46. Cutting the same hypothetical resistance map of 3000x3000 pixels into 250x250 pixel tiles, results in a grid of 12x12 tiles, or 144 tiles. According to Table S1 in Appendix S3, each 1000x1000 tile with a 75% buffer takes 27 minutes and 28 seconds to process in all four directions on our computer. The total serial processing time for all 9 tiles is 4 hours and 7 minutes. The 250x250 pixel tiles with a 200% buffer each take 5 minutes and 54 seconds to process. The total serial processing time for all 144 tiles is 14 hours and 10 minutes. Because of the effect of buffer size on total processing time, the computing time required to produce the 250x250@200% mosaic is more than 3 times longer than that for the 1000x1000@75% mosaic.

Because large landscapes can be tiled and treated separately, it may be fruitful to run multiple instances of Circuitscape in parallel if the computer used has enough available physical memory and processor cores. Using the previous example, each tile of the 1000x1000@75% mosaic, with a calculation area of 2500x2500, requires about 5.62 GB of memory for processing. On a computer common at the time of writing of this manuscript with 16 GB of ram and 12 processor cores at 2.6 GHz, it is possible to process 3 tiles at the same time. Without taking into account other parameters such a simultaneous disk access, which could slow down the processing time, this reduces the total theoretical processing time from 4 hours and 7 minutes to about 1 hour 22 minutes. The 250x250@200% mosaic, with a calculation area of 1250x1250 pixels, uses only about 1.58 GB of memory. It is possible to process 10 at the same time in 16 GB of physical memory. Again, without taking into account other factors that could affect processing performance, the theoretical processing time decreases from 14 hours 10 minutes to around 1 hour 25 minutes. The processing times are now virtually the same on the computer used in this example. Given the seams prominence of the final mosaics as reported in Figure S2 in Appendix S1, larger tiles with smaller buffers are preferable on computers with few processor cores and with limited memory. But on a faster computer with a lot of physical memory and many processor cores, smaller tiles with large buffers might be preferable.

In a larger multi-processing environment such as a university computing cluster, it is possible to process hundreds of tiles simultaneously. It would be possible to calculate connectivity for very large areas, such as whole countries or even continents in a relatively short amount of time using the tiling method presented here.

### Measuring processing time and peak memory usage

The processing time was measured using data from the Montérégie resistance map for all the sizes listed in Table S1 in Appendix S3. We processed each tile three times and averaged the processing times returned by Circuitscape. To measure peak memory usage, we used the Valgrind dynamic program analysis tool while running Circuitscape. The processing time and memory usage was calculated on an early 2013 Mac Pro with two 2.6 GHz six-core Intel processors and 64 GB of physical memory running Ubuntu Linux 12.10 64 bits.

## Figures

**Calculation area size and memory usage**


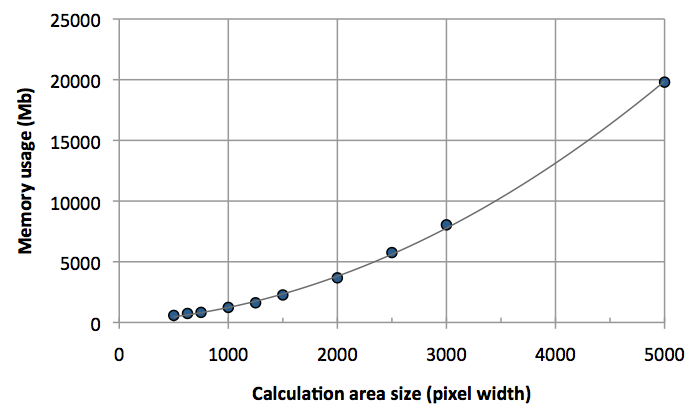


Figure S1: calculation area size and memory usage.

**Pixel count and memory usage**


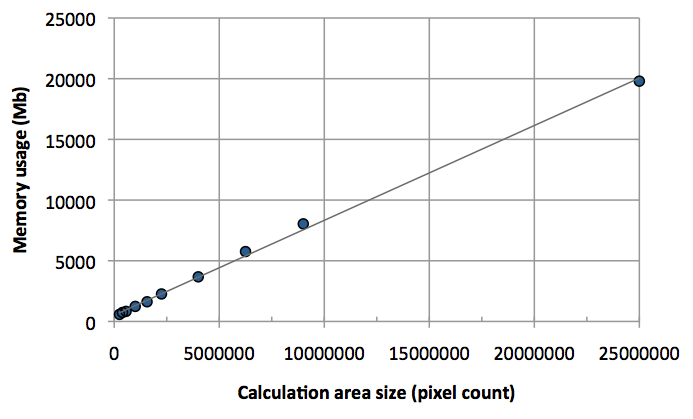


Figure S2: total pixel count of the calculation area and memory usage.

**Calculation area size and processing time**


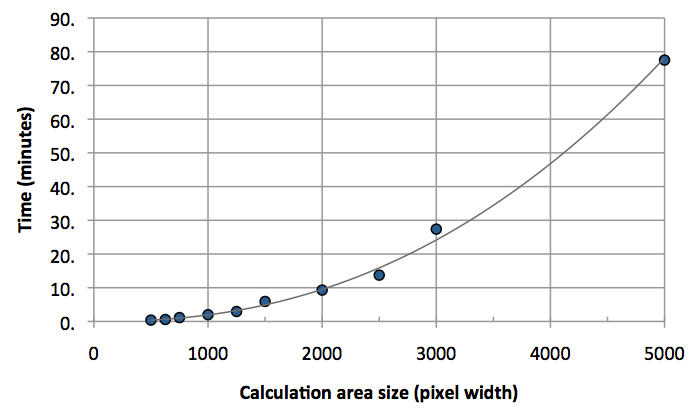


Figure S3: calculation area size and processing time.

## Tables

**Table of processing time and memory usage**

| **Tile size**  **(pixels)** | **Buffer size (% / pixels)** | **Calculation area (pixels)** | **Processing time (all 4 directions)** | **Peak memory usage (MB)** |
| --- | --- | --- | --- | --- |
| 250x250 | 50% / 125 | 500x500 | 0:48 | 579.77 |
| 250x250 | 75% / 188 | 626x626 | 1:10 | 739.37 |
| 250x250 | 100% / 250 | 750x750 | 2:18 | 830.01 |
| 250x250 | 200% / 500 | 1250x1250 | 5:54 | 1620.16 |
| 500x500 | 50% / 250 | 1000x1000 | 4:00 | 1238.75 |
| 500x500 | 75% / 375 | 1250x1250 | 5:54 | 1620.16 |
| 500x500 | 100% / 500 | 1500x1500 | 12:52 | 2264.83 |
| 500x500 | 200% / 1000 | 2000x2000 | 18:36 | 3676.41 |
| 1000x1000 | 50% / 500 | 2000x2000 | 18:36 | 3676.41 |
| 1000x1000 | 75% / 750 | 2500x2500 | 27:28 | 5756.27 |
| 1000x1000 | 100% / 1000 | 3000x3000 | 54:46 | 8041.51 |
| 1000x1000 | 200% / 2000 | 5000x5000 | 2:04:22 | 19797.72 |

Table S1: Processing time and memory usage for each tile and buffer size combinations and the resulting calculation area size.
